# Development of a Neurodegenerative Disease Gait Classification Algorithm Using Multiscale Sample Entropy and Machine Learning Classifiers

**DOI:** 10.3390/e22121340

**Published:** 2020-11-25

**Authors:** Quoc Duy Nam Nguyen, An-Bang Liu, Che-Wei Lin

**Affiliations:** 1 Department of Biomedical Engineering, College of Engineering, National Cheng Kung University, Tainan City 701, Taiwan; namduy1012@gmail.com; 2 Department of Neurology, Hualien Tzu Chi Hospital, Buddhist Tzu Chi Medical Foundation and Tzu Chi University, Hualien 970473, Taiwan; liuabpaper@yahoo.com.tw; 3 Department of Biomedical Engineering, College of Engineering/Medical Device Innovation Center, National Cheng Kung University, Tainan City 704, Taiwan

**Keywords:** Neurodegenerative disease, multiscale sample entropy, gait analysis

## Abstract

The prevalence of neurodegenerative diseases (NDD) has grown rapidly in recent years and NDD screening receives much attention. NDD could cause gait abnormalities so that to screen NDD using gait signal is feasible. The research aim of this study is to develop an NDD classification algorithm via gait force (GF) using multiscale sample entropy (MSE) and machine learning models. The Physionet NDD gait database is utilized to validate the proposed algorithm. In the preprocessing stage of the proposed algorithm, new signals were generated by taking one and two times of differential on GF and are divided into various time windows (10/20/30/60-sec). In feature extraction, the GF signal is used to calculate statistical and MSE values. Owing to the imbalanced nature of the Physionet NDD gait database, the synthetic minority oversampling technique (SMOTE) was used to rebalance data of each class. Support vector machine (SVM) and *k*-nearest neighbors (KNN) were used as the classifiers. The best classification accuracies for the healthy controls (HC) vs. Parkinson’s disease (PD), HC vs. Huntington’s disease (HD), HC vs. amyotrophic lateral sclerosis (ALS), PD vs. HD, PD vs. ALS, HD vs. ALS, HC vs. PD vs. HD vs. ALS, were 99.90%, 99.80%, 100%, 99.75%, 99.90%, 99.55%, and 99.68% under 10-sec time window with KNN. This study successfully developed an NDD gait classification based on MSE and machine learning classifiers.

## 1. Introduction

Neurodegenerative disease (NDD) is the process of neuronal death in different nervous system areas, resulting in the loss of structure and functions for neurons. Many NDDs exist, including Parkinson’s disease (PD), Huntington’s disease (HD), amyotrophic lateral sclerosis (ALS). The prevalence of PD is approximately 1% of the population older than 60 [1,2], 15% of patients have a family history [3], and 10% have a mutation in genes [4]. HD is an inherited disorder and disease usually begin at around 30 to 50 years old [5,6,7] and the most common symptoms of the body are uncontrollable movements called chorea, jerking, and abnormal posturing [8,9]. In addition, ALS is a chronic and fatal form of motor neuron disease and it is the third most common NDD which is the incidence rate is 2.7/100,000 people/year [10].

Gait analysis is a method that identifies biomechanical abnormalities in the gait cycle and can identify potential flaws that could lead to injuries, inefficiencies, and inconveniences [8]. The gait analysis application can help NDD patients diagnosed at an early stage by collecting data such as the gait force (GF) signals and the gait cycle patterns (e.g., stride times, swing times, stand times, stride-to-stride measures of contact times). From the results of the gait analysis, researchers can develop suitable solutions to minimize NDD progression.

Plenty of studies have used the Internet available database PhysioNet Gait in Neurodegenerative Disease Database (PGNDD) website by Hausdorff et al. [9] to develop NDD classification algorithms by different features such as gait cycle patterns from GF signals [11]; Fourier transform of sequences [12], statistics values [13], RQA parameters [14], and fuzzy recurrence plot [15] of the GF signals. Besides, machine learning and deep learning models commonly used in classifying NDD data are support vector machine (SVM) [11,12,13,14,15] and also have other models as *k*-nearest neighbors (KNN) [13], multi-layer perceptron (MLP) [13], and probabilistic neural network (PNN) [14], least squares SVM (LS-SVM) [15]. In addition to the machine learning algorithm, state of the art convolutional neural network (CNN) is used for NDD gait classification [16]. Regarding the cross-validation, leaving one out cross-validation (LOOCV) approach was often used to validate the training process. Representative literature of NDD gait classification articles is summarized in Table 1.

In recent years, the processing of signals from physiological systems, such as the brain, heart, and muscles, has become commonplace. The signals from these organs contain information that allows researchers to detect its abnormal. However, the processing of biomedical signals is becoming more and more complex and requires extracting information from data converted from visual observations; further processing is a necessity. Entropy concept is used in many scientific fields such as information theory, chaos theory, statistical mechanics, and many other fields [17]. Entropy is considered to be a measure of the turbulence present in the observed environment [18]. If the disturbance level is low, then the systems become organized. In contrast, the level of disturbance is high, and then the observed environment lacks stability. Several entropy methods have been developed in previous studies, such as regional entropy, multiscale entropy (MSE) [19,20], approximate entropy, sample entropy, cross multiscale entropy [21,22], permutation entropy [23,24], and time-shift multiscale entropy [25]

In addition, entropy method is quite popular in electroencephalogram (EEG) signal [24,25,26,27], electrocardiogram (ECG) signal [28,29], and electromyography (EMG) signal [30,31]. Mizuno et al. and Labate et al. used MSE to analyze the complexity of signaling in patients with Alzheimer’s disease [27,32]. Other studies by Ouyang et al. and Zeng et al. also used the multiscale permutation entropy analysis and spatial-temporal permutation entropy applied to EEG signals to detect differences in the seizure-free, pre-seizure, and seizure states in brain activity [23,24]. Lu et al. extracted successive entropy values in quantitative EEG signals over time known as dynamic entropy-based patterning, it is possible to achieve subject-independent emotion recognition [25]. Mahajan et al. introduced a new unsupervised machine learning model and used multiscale sample entropy (MSE) and kurtosis as features to identify independent eye-blinking artifacts [33]. In addition, Platiša et al. used MSE to measure the complexities of the cardiorespiratory system over the cardiac interval [21] and Roldan et al. used MSE to analyze the *f*-waves may provide early prediction of atrial fibrillation recurrence after electrical cardioversion in ECG signals [28]. Zhao et al. applied a threshold-based sample entropy to suppress the influence of ectopic beats for heart rate variability analysis [29]. Regarding applying the entropy theorem in EMG analysis, Trybek et al. and Qin et al. extracted the MSE features to evaluate the surface electromyography (sEMG) signals [30,31]. In summary, entropy is widely applied in the physiological signal analysis especially EEG/ECG/EMG. Furthermore, to extract entropy features and integrate with machine learning/deep learning makes complicate physiological signal more feasible [34,35,36].

However, existing literature using entropy in NDD gait analysis is rare. To name a few, Liu et al. and Yu et al. used multiscale approximate entropy (MAE) [37] and symbolic entropy [38] to analyze ground reaction force on both feet and calculate complexity of human gait. Liao et al. applied the multi-resolution entropy analysis of stance time fluctuation to investigate the gait asymmetry [39]. Ren et al. extracted the phase synchronization and conditional entropy features from gait cycle patterns to differentiate gait pattern from healthy control (HC) to that of PD/HD/ALS. Classification results were poor except for HC vs. HD [35]. Wu et al. computed the approximate entropy, normalize symbolic entropy, and signal turns count to classify different gait pattern from HC and PD and best accuracy is 84.48% [34].

The literature survey shows that to use entropy for classification HC and any type of NDD is a potential research topic, especially to extract the feature from raw data of gait signal. Therefore, the aim of this study is to develop an NDD gait classification algorithm for screening patients with NDDs based on their GF signals using entropy features. Entropy is good at evaluating the turbulence or chaotic level of system/signal and it may be helpful to develop NDD gait classification algorithms by integrating entropy related features and machine learning algorithms.

## 2. Materials and Methods

### 2.1. PhysioNet Gait in Neurodegenerative Disease Database

The PhysioNet Gait in Neurodegenerative Disease Database (PGNDD) [9] provided by Hausdorff et al. was adopted in this study. The dataset from PGNDD consisted the GF signals of 64 subjects, including 16 HC subjects, 15 PD subjects, 20 HD subjects, and 13 ALS subjects. The demographics of subject in PGNDD is shown in Table 2. The PGNDD database includes two types of recorded data: (1) raw data of the GF signals and the (2) gait cycle patterns derived from the GF signals (The gait cycle patterns from the GF signals comprise the stride times, swing times, stand times, stride-to-stride measures of contact times). Only the GF signals were used in the study because the purpose of this study is to develop the NDD gait classification using entropy related features. Entropy features need large amount of data to calculate [40,41], and the number of data samples of gait pattern is much less than the data sample of GF signals. Hence the gait patterns are not considered to use for generating entropy features.

GF signals of each subject was required to walk without assistive devices or a wheelchair for 5 min. The sampling frequency of GF signal in PPGNDD was 300 Hz. The raw data of the GF signal were obtained by applying resistors of force-sensitive in the insole, and the output comprised the values proportional to the force of the foot. The sole was made from a manila folder by following the contour of the foot and then cutting on the mark. One sensor was located on the front part of the insole under the toe and ankle, and the other was on the opposite end under the heel.

**Figure 1:**
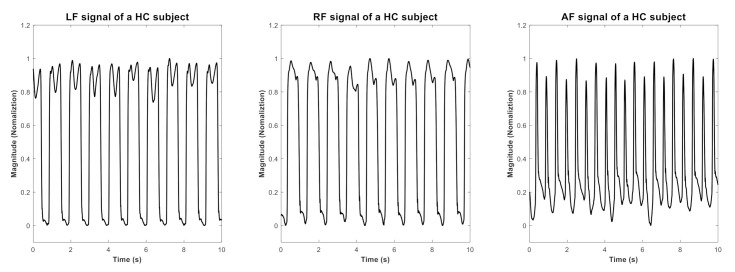
An example illustrating the LF, RF and AF signal of HC subject in 10-sec window length.

The GF signals comprise the left foot (LF) and right foot (RF) signal. A combination: average foot (AF) signals defined in this study was derived from averaging the LF and RF signals by using the Equation (1). and depicts in Figure 1:

(1) $$AF\;=\;\left(LF+RF\right)/2$$

### 2.2. Neurodegenerative Disease Gait Classification Algorithm Using Entropy Features and Machine Learning Algorithms

**Figure 2:**
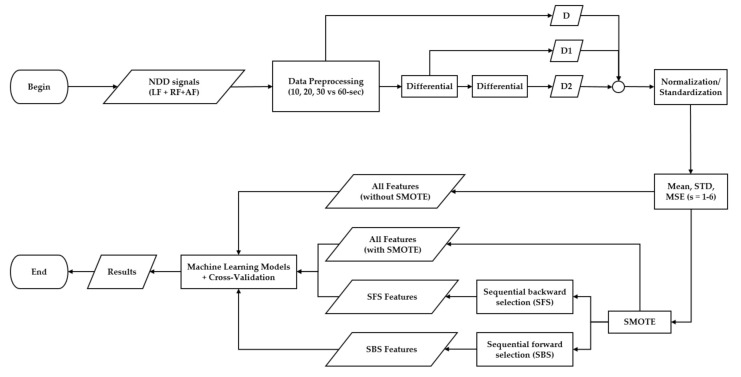
Flowchart of the proposed algorithm process from the input stage to the classification stage.

The proposed NDD gait classification algorithm using entropy features is shown in Figure 2. The proposed algorithm consists of data preprocessing, feature extraction, data augmentation, feature selection, and machine learning models. In the first step of data preprocessing, the LF/RF/AF are used as the input data (denoted as set ***D***). A new set of input data ***D1*** and ***D2*** are created by taking one and two times of differential using Equation (2) on ***D***. After the above computations, the original three-dimensional input data ***D*** (LF/RF/AF) will be extended to nine-dimensional input data including three-dimensional input data from ***D*** (LF/RF/AF), three-dimensional input data from ***D1*** (take one time differential on ***D***, which denoted as LF1, RF1, and AF1), three-dimensional input data from ***D2*** (take one differential of ***D1***, which denoted as LF2, RF2, and AF2). The second step of data preprocessing is to segment input data (***D***, ***D1***, and ***D2***) into consecutive window data with 50% overlap (denoted as the input window data). In the third step of data preprocessing, data window which includes obvious artifact are excluded in the last step of data preprocessing. In the last step of data preprocessing, ***D***, ***D1***, and ***D2*** were normalized by using Equations (3) and (4).

(2) $$X^{\prime }=\mathrm{diff}\left(X\right)=\left\{x_{2}-x_{1},\;x_{3}-x_{2},x_{4}-x_{3},\ldots ,x_{n}-x_{n-1}\right\},$$

(3) $$X^{\prime }=\frac{X-\min\left(X\right)}{\max\left(X\right)-\min\left(X\right)}$$

(4) $$X^{\prime }=\frac{X-µ}{\sigma },$$

where *µ* is average and σ is the standard deviation.

In the feature extraction, mean, standard deviation (STD), and multiscale sample entropy (MSE) features (*s* = 1–6) were applied on the nine-dimensional input window data ***D*** (LF/RF/AF), ***D1*** (LF1/RF1/AF1), and D2 (LF2/RF2/AF2). In each dimension of the input window data, eight features mean, STD and 6 features of MSE (*s* = 1–6) will be computed, hence 72 features (eight features derived from each dimension of input window data and there are a total of nine dimensions in input data) will be obtained from each input window data.

For the data augmentation steps, in order to deal with the data imbalanced nature of the PGNDD (16 HC subjects, 15 PD subjects, 20 HD subjects, and 13 ALS subjects) the synthetic minority oversampling technique (SMOTE) [42,43] process was applied to solve the problem of imbalance that exists in the database. Besides, sequential forward selection (SFS) and sequential backward selection (SBS) were applied to reduce the dimensions of the measured features and select features that contribute the most without reducing the accuracy [44,45,46] in the feature selection. Finally, the selected features by SFS/SBS are input into machine learning models as KNN/SVM models for classification.

#### 2.2.1. Data Preprocessing

**Figure 3:**
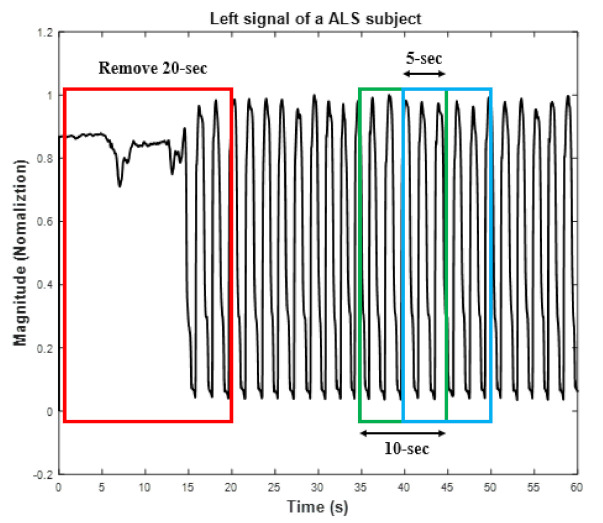
An example illustrating to remove the first 20-sec of recording and windowing in data preprocessing.

**Figure 4:**
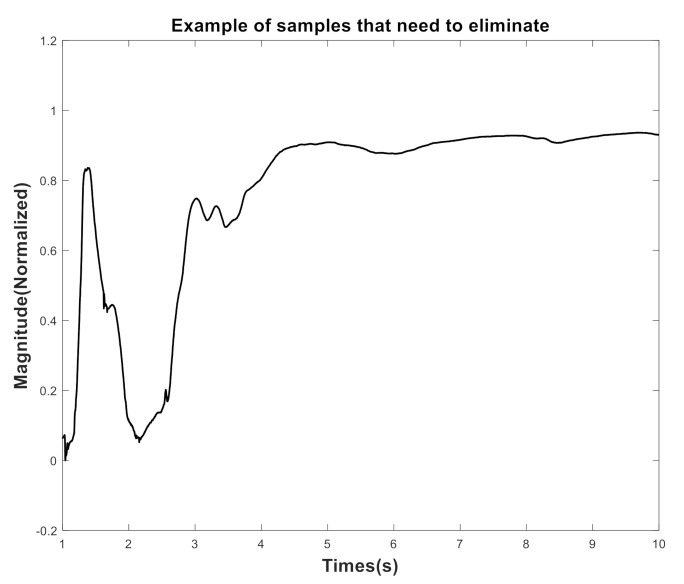
An example illustrating the artifact window data that needed to be discarded.

The original GF signals were collected for 5 min per subject. The first 20-sec of data were removed to eliminate the influence of each subject’s initial walking interval since it is usually not a normal walk pattern at beginning of data recording (one example can be seen from the red rectangle box of Figure 3). In the proposed algorithm, the rectangular window function is applied to split the input data (LF/RF/AF/LF1/RF1/AF1/LF2/RF2/AF2) into consecutive input window data with 50% overlap and various window length (10/20/30/60-sec). The green and blue rectangle box in Figure 3 depicts an example of the window process under 10-sec window with 50% overlap. To ensure that all input data are not affected by external factors altering the signal shape, we visually examined each one and directly discarded them. Figure 4 is an example illustrating an input data with an artifact to be removed.

The method of dividing the data using overlapping windows was adopted by the definition in [16] and the number of GF signals samples obtained from the process can be depicted in Equation (5)

(5) $$n=\left(\frac{\left(l-TW\right)}{d}+1\right)\times T,$$

where *l* (sec) is the time length of the signal, *TW* is the time window length (10/20/30/60-sec), *d* (sec) is the overlapping between consecutive windows, and *T* is the total subjects in each group.

#### 2.2.2. Feature Extraction

##### Statistical Features

In this research, the statistical features including the mean and standard deviation (STD) were applied to extract features from ***D/D1/D2*** as shown in Equations (6) and (7).

(6) $$\mathrm{Mean}=\frac{1}{N}\overset{N}{\underset{i=1}{\sum }}x_{i}$$

(7) $$\mathrm{STD}=\sqrt{\frac{1}{N}\overset{N}{\underset{i=1}{\sum }}{\left(x_{i}-µ\right)}^{2}}$$

where $X=\{x_{1},{\;x}_{2},{\;x}_{3},\;\ldots ,{\;x}_{N}$} is input data from ***D/D1/D2*** (LF/RF/AF/LF1/RF1/AF1/LF2/RF2/AF2) with *N* samples.

##### Multiscale Sample Entropy (MSE)

**Figure 5:**
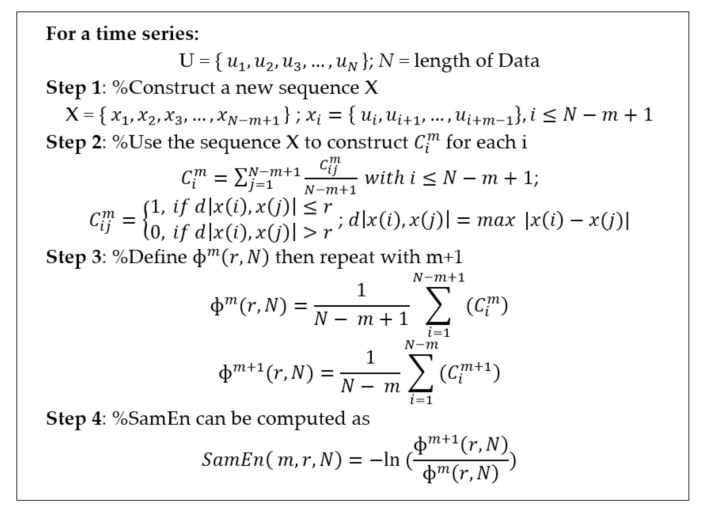
Sample entropy pseudocode.

**Figure 6:**
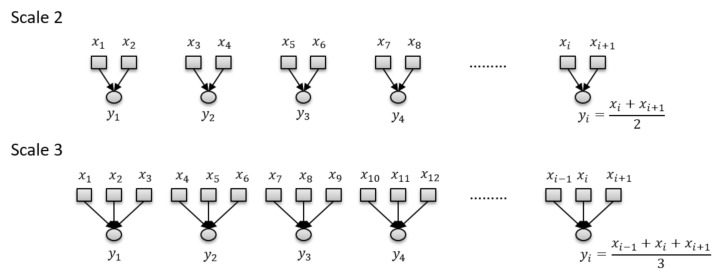
Illustration of the scale generation stage in the multiscale sample entropy algorithm.

Entropy is a measure that describes the amount of regularity and the unpredictability of fluctuations over time-series data. Entropy has a higher value if the complicated level of sequences is large, and vice versa. Sample entropy method is one of the representative entropy measures and has been used to diagnose a diseased state by assessing the complexity of physiological time-series signals [47,48]. Sample entropy values are dependent on three parameters: the length of the embedding dimension *m*, tolerance *r*, and length of signal *N* [49]. The sample entropy algorithm is explained in Figure 5. Both parameters *m* and *r* greatly influence sample entropy values. The parameters *m* and *r* were set to 3 and 0.2 respectively in this study [49,50]:

Multiscale sample entropy (MSE) is an extension of the standard sample entropy method and is used to evaluate the signal complexity over a time-scale range [50]. It expands the sample entropy method to various time scales to provide an additional perspective [49]. Like the sample entropy measure, the goal of MSE is to assess the complexity of a time series [40]. The main reason to use a multiscale approach is to search for more information across various time scales and investigate the relations between MSE time scale and NDD GF signal. The MSE method principle involves reducing the number of data points in a time series using the operation while the scale increases. The process of generating scale for a time series $X=\{x_{1},\;x_{2},\;x_{3},\;\ldots ,\;x_{N}$} on MSE computation is described in Figure 6 and represented as Equation (8) [49,50]. Finally, the MSE values can be obtained by using various *s* in Equation (8). The parameter of *s* is set from 1 to 6 in this study.

(8) $$y_{j}^{s}=\frac{1}{s}\overset{js}{\underset{i=\left(j-1\right)s+1}{\sum }}x_{i},\;1\leq j\leq \frac{N}{s},\;\mathrm{where}\;s\;\mathrm{is}\;\mathrm{scale}$$

For each input window data in the feature extraction process, eight features including mean, standard deviation, and MSE (*s* = 1–6) values were applied night-dimensional input data: ***D*** (LF/RF/AF), ***D1*** (LF1/RF1/AF1) and ***D2*** (LF2/RF2/AF2). There will be 72 features (noted as F1–F72) generated for each input data window during feature extraction. Description of notation F1–F72 can be found in Table 3. For example, F1–F8 represents the feature derived from LF signal (*i* = 1) and F9-F16 represents the feature derived from LF signal (*i* = 2).

#### 2.2.3. Synthetic Minority Oversampling Technique (SMOTE)

The database adopted in this study [9] is considered imbalanced because it has an unequal number of instances (samples or data points) for different NDD. A class with a relatively smaller number of samples is considered a minority class, whereas a class with a relatively larger number of samples is called a majority class. When data are highly imbalanced, it significantly affects the classification accuracy. One way to solve this problem is to oversample the minority layer data, which can be done by duplicating the samples from the minority class in the training dataset. The SMOTE was proposed to tackle the issue of class imbalance [42,51,52]. The SMOTE is a widely used oversampling technique that performs better than simple oversampling by creating synthetic minority class samples. This technique is based on the closest neighbors assessed by Euclidean distance between data points in a feature space. The SMOTE works by selecting examples close to the feature space, drawing a line between the examples in the feature space, and taking a new sample at a point along that line. The formula to generate synthetic data using the SMOTE is expressed as:

(9) $$x^{\prime }=x+\mathrm{rand}\left(0,1\right)\times \left|x-x_{k}\right|,$$

where *x’* denotes an augmented new example, *x* is an example from the minority class, $x_{k}$ indicates one of the *k*-nearest neighbors from *x*, and rand (0, 1) represents a random number between 0 and 1. In this study, we assume an imbalance in the database can affect the accuracy of the proposed method, the SMOTE was used to address this issue [42,43].

#### 2.2.4. Sequential Feature Selection

Sequential feature selection techniques are feature searching algorithms used for reducing the original dimensions of the measured features (predictor variables) by selecting a subset to create a model. Algorithms select the most relevant features that optimally model the response, improve computational efficiency, and reduce the generality error of the model. The techniques have two variants: sequential forward selection (SFS) [45] and sequential backward selection (SBS) [46]. The purpose of using SFS/SBS is to increase efficiency and reduce the number of computations of the machine learning classification model at a later stage.

##### Sequential Forward Selection (SFS)

**Figure 7:**
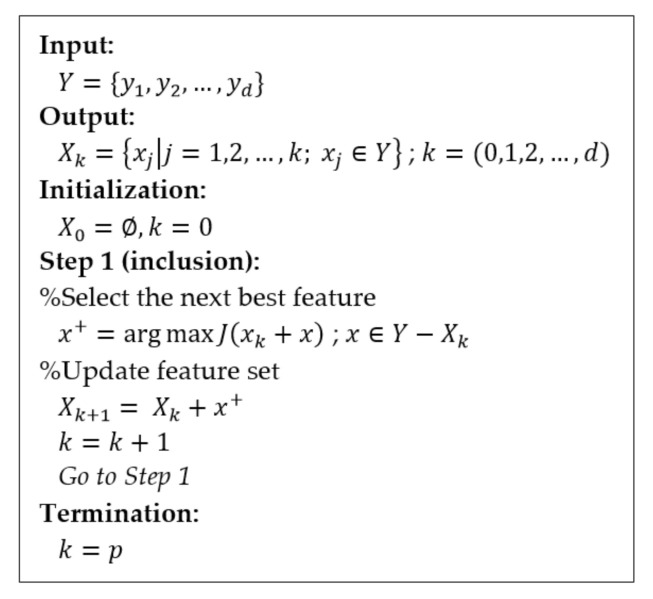
Pseudocode of SFS.

With SFS, features are sequentially added to an empty candidate set and tested at each step until the addition of further features no longer improves the misclassification rate of the classification model, and then the process stops [45,46]. The SFS is a search algorithm that determines an optimal feature extraction set by sequentially adding a single feature from an empty set until it increases the value of the objective function. The pseudocode for the SFS algorithm is given in Figure 7 [44,45,46]. In the input stage, the SFS algorithm takes *d*-dimensional features as input. In the beginning, the algorithm initializes with an empty set ∅ (“null set”) so that k=0 (where k is the length of the subset). In addition, $x^{+}$ is the maximizing feature in the criterion function, which has the best classifier performance and is added to X*_k_* in the first step. This procedure repeats until the termination criterion is satisfied. For the termination, the procedure only stops when the number of features added to the feature subset X*_k_* reaches the feature subset of size *k* obtaining the number of desired features *p*. The SFS returns a subset of features in the output, where the number of selected features is *k* (*k* < *d*).

##### Sequential Backward Selection (SBS)

**Figure 8:**
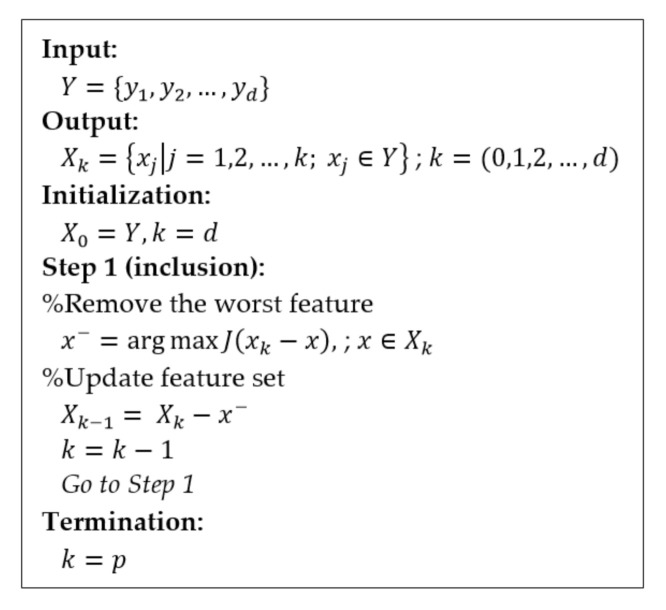
Pseudocode of SBS.

In contrast to SFS, the SBS technique begins with the full candidate set and then iteratively removes the least contributing feature step-by-step [46]. The SBS is an iterative algorithm that considers all features for inclusion in the final feature subset that works in the opposite direction from SFS. The pseudocode for the SFS algorithm is provided in Figure 8 [44,45,46]. In the input stage, the SBS takes the whole feature set as input, and the algorithm initializes with the given feature set so that k=d. In the first step, a feature $x^{-}$ is removed from the feature subset $X_{k}$. Moreover, $x^{-}$ is the maximizing feature in the criterion function, which has the best classifier performance and is removed from $X_{k}$. This procedure is repeated until the termination criterion is satisfied. For the termination, the procedure only stops when the number of features removed from the feature subset $X_{k}$ reaches the feature subset of size k containing the number of desired features *p*. For the output, SBS returns a subset of features: the number of selected features *k*, where k<d.

#### 2.2.5. Machine Learning Model

After completing the feature extraction, data augmentation, and feature selection phase. The classification based on machine learning models, support vector machine (SVM) technique, and *k*-nearest neighbors (KNN) technique were used in this study

##### Support Vector Machine (SVM)

The SVM is a supervised machine learning algorithm that discriminates the classifier formally defined by a separating hyperplane [52]. After training, the output is an optimal hyperplane that can categorize new examples. The SVM was initially formulated from the problem of the quadratic optimization of Vapnik’s statistical theory in which the surface error is free of local minima and has a global optimum [53]. The SVM’s main concepts are using a kernel function and then constructing an optimum separation hyperplane between the two classes in the transformed space to transform the input data space into higher-dimensional data space [52,53]. The hyperplane is achieved in the SVM algorithm by optimizing the margin classification for separable patterns in an *m*-dimensional space. The hyperplane must linearly separate the two classes $\left\{+1,-1\right\}$ on either side of the hyperplane. The equation for the decision surface (hyperplane) is represented as Equation (10).

(10) $$w^{T}x+b=0,$$

where w is the adjustable weight vector and b is the bias of the hyperplane. The linearly separable classes can be represented as Equation (11).

(11) $$w^{T}x+b\leq 0\;\mathrm{for}\;d_{i}=-1,\;w^{T}x+b>0\;\mathrm{for}\;d_{i}=+1$$

The optimization problem can be mapped to the quadratic optimization problem with global minimum and linear constraints [52].

SVM algorithms are built to solve the binary classification problem, with only two classes. Models work with the problem of having two classes called binary classifiers [54]. A natural way to extend these models to apply to multi-class classification problems, which have many different classes, is to use multiple binary classifiers and techniques like one-vs-one [55]. In a one-on-one, multiple binary classifiers are built for each pair of classes. For example, the first set classifies classes 1 and 2, the second set classifies classes 1 and 3, and so on. When data is entered, it builds all the binary classifiers as in the above example. The result can be determined according to the class in which the data are most divided (major voting). 

##### *K*-Nearest Neighbors (KNN)

The KNN method is also an essential supervised learning algorithm in machine learning, and the type of KNN is lazy learning because this algorithm does not learn anything from the training data [56]. The KNN algorithm assigns a category to observations in the test dataset by comparing them to the training dataset observations [23]. In this algorithm, an object is classified according to the number of neighbors that have the same class around them and are assigned to the most popular class among them. If *k* = 1, then the object is assigned to the class of its nearest neighbor, and fine KNN was used in this study [57].

Further, KNN classification has two stages: the determination of the nearest neighbors and the determination of the class of those neighbors [13]. With a training dataset D comprising $\left(x_{i}\right){\;}_{i\in \left[1,\left|D\right|\right]}$ training samples, a set of features *F* is extracted from training data D, and any numeric features are normalized to the range [0,1]. Each training example is labeled with a class label $y_{j}\in Y$. The objective is to classify an unknown example q. For each $x_{i}\in D$, the distance between q and $x_{i}$ is calculated as:

(12) $$d\left(q,x_{i}\right)=\;\sum_{f\in F}w_{f}\delta \left(q_{f},x_{\mathrm{if}}\right).$$

A large range of possibilities exists for this distance metric. A basic version for continuous and discrete attributes is as follows:

(13) $$\delta \left(q_{f},x_{\mathrm{if}}\right)=\left\{\begin{array}{c} 0,\;f\;\mathrm{discrete}\;\mathrm{and}\;q_{f}=x_{\mathrm{if}}\\ 1,\;f\;\mathrm{discrete}\;\mathrm{and}\;q_{f}\neq x_{\mathrm{if}}\\ \left|q_{f}-x_{\mathrm{if}}\right|,\;f\;\mathrm{continuous} \end{array}\right..$$

#### 2.2.6. Validation Technique

Cross-validation is a statistical method to access and compare learning algorithms by dividing data into two groups: training set and validation set [52]. Training and validation sets must repeat in consecutive loops so that each data can have an opportunity of being validated [58]. There are two main purposes for this technique: the first purpose is to quantify the generalizability of an algorithm. The second purpose is to evaluate the performance of two or more different algorithms and discover the best algorithms. The *k*-fold cross-validation was used in this study. *k*-folds are established by first partitioning the data points [59]. Consequently, *k* iterations of training and validation are carried out that within each iteration, a different fold of the data points is applied for validation while remaining (*k* − 1) folds are utilized for learning. 10-fold cross-validations were applied in this study.

## 3. Results

The results are presented in three experiments: (1) classification of the HC group and each disease from NDD groups (two-class); (2) classification of any two of the disease groups from NDD groups (two-class); (3) classification of the HC and each disease in the NDD groups (multi classes). Each experiment presents the classification accuracy under various conditions such as using SMOTE data augmentation or not, using data selection techniques (SFS/SBS) or not, and different classifiers (KNN and SVM). The computations were conducted by MATLAB R2019a software. Table 4 reveals the number of samples used in this study under various time window, initially extracted samples (IES) indicates the samples extracted based on Equation (5). Verified samples (VS) indicates the number of samples after visual checking the signal quality. The number of samples after SMOTE data augmentation is shown in Table 4.

### 3.1. Classification of the Healthy Control Group and Each Disease from Neurodegenerative Diseases Groups (Two-Class)

Table 5 shows the classification results of the tasks in the first experiment for (HC vs. PD), (HC vs. HD), and (HC vs. ALS) for the 10, 20, 30, and 60-sec window lengths. For each selection method, each classification model (KNN or SVM) associated with each classification task (e.g., HC vs. ALS) in different window lengths has a different accuracy. Overall, at windows as small as 10 and 20-sec, the highest classification accuracy was almost 100% on all three tasks. However, at windows as 30 and 60-sec, the classification accuracy decreases gradually and the highest accuracy is 99.55% (30-sec, SVM, All features, with SMOTE), 99.70% (60-sec, KNN, SFS features), and 99.85% (30-sec, SVM, All features, with SMOTE) corresponds to (HC vs. PD), (HC vs. HD) and (HC vs. ALS). The classification accuracy of all features with and without using SMOTE is not much different. The results from the KNN model seem higher than the SVM model.

### 3.2. Classification of Any Two Diseases Groups from Neurodegenerative Disease Groups (Two-Class)

In the second experiment, the same algorithm techniques as the first experiment were conducted. The difference is to classify diseases among the NDD groups. The purpose is to provide the intra-class separation of diseases in the NDD groups regarding whether they are easy to differentiate through GF signal features. Table 6 lists the classification results for (PD vs. HD), (PD vs. ALS), and (HD vs. ALS) for 10, 20, 30, and 60-sec window lengths. In general, similar to the first experiment, with windows as small as 10 and 20-sec, the classification accuracy is very high, at 100% (20-sec, KNN, all features, with SMOTE), 100% (20-sec, KNN, SFS features), and 99.83% (10-sec, SVM, all features, without SMOTE) corresponds to (PD vs. HD), (PD vs. ALS), and (HD vs. ALS). In contrast, windows at 30- and 60-sec have a slight decrease in accuracy and the highest accuracy is 99.70% (60-sec, KNN, SFS features), 100% (60-sec, KNN, SBS features), and 99.62% (60-sec, SVM, all features, without SMOTE) corresponds to (PD vs. HD), (PD vs. ALS), and (HD vs. ALS). The classification accuracy of all features with and without using SMOTE is also not much different. The results from the KNN model also seem higher than the SVM model.

### 3.3. Classification of the Healthy Controls and Each Disease in the Neurodegenerative Disease Groups (Multi-Class)

In the last experiment, the multi-class classification between HC vs. PD vs. HD vs. ALS was conducted. The procedure and algorithms used in the feature extraction stage are similar to those of the first and second experiments. Table 7 presents the multi-class classification accuracy of 10, 20, 30, and 60-sec window lengths. The highest classification accuracy is 99.73% (SFS features, KNN), 99.77% (all features, with SMOTE, KNN), 99.15% (all features, with SMOTE, SVM), and 99.69% (SBS features, KNN) correspond to 10-, 20-, 30- and 60-sec window lengths. For all features with and without using SMOTE, the difference in classification accuracy is not clear in 10- and 20-sec window lengths. However, there is a clear difference in the KNN model, 96.98% vs. 98.53% at the 30-sec, and 94.40% vs. 96.41% at 60-sec.

## 4. Discussion

This section presents the discussion of the factors that contribute to the novelty and precision of the proposed algorithm. These include the transformation of the original GF signal using Equation (2) to generate two new signal types: window lengths (10-/20-/30-/60-sec), SMOTE method, sequential selection methods (SFS and SBS), and classification models (KNN vs. SVM). Finally, we compare our results with the existing studies.

### 4.1. Contribution of Combining Entropy Features and Feature Selection in NDD Gait Classification

In Table 1, many previous studies have also used NDD datasets [9] with different feature extraction approaches, such as FRP [15], GLCM [15], or feature extraction using Fourier transform signals on the frequency domain [12] or statistical values as features [13]. Experiment results of this study reveal that the statistical and MSE features mentioned derived from GF (***D***) and taking one (***D1***) and two times (***D2***) of differential can achieve satisfactory classification results both in two-class or multi-class NDD gait classification. Although the feature generation of the proposed algorithm leads to an increase in the number of features. The computation complexity can be reduced by effective feature selection (SFS/SBS) in this study.

### 4.2. Effect of Time Window Length in NDD Gait Classification

From Table 5, Table 6 and Table 7, with increasing window lengths, the accuracy of the method decreases. However, a decrease in the classification accuracy as the window length increases does not indicate that this method is not good for large window lengths (60-sec). Patients may not be able to repeatedly walk alone for 30- or 60-sec without needing help. The diagnosis becomes a burden to the patient if the patient must walk too long or too often. Therefore, using a small window length is convenient. The proposed method does not require a too-high calculation ability in window lengths of 10 or 20-\ sec. Compared to the existing literature, the proposed method can achieve a high accuracy on NDD gait classification under a short time window.

### 4.3. Effect of SMOTE Data Augmentation

Due to the clinical features and the rarity level, the number of patients in each class is different. ALS patients are the rarest, so this imbalance affects the training and accuracy of the whole process. The SMOTE method was suggested to use if the difference between the number of each layer is not too much. Based on observation of Table 4, the quantity difference in each class of the NDD database was not too much. Table 5, Table 6 and Table 7 show that a slight increase in accuracy can be seen in the majority of classification tasks between with and without SMOTE. This shows that the method can help improve accuracy where the number of samples in each class is not too much, especially in 30- or 60-sec time window length.

### 4.4. Effect of Sequential Feature Selection Methods

The purpose of using this method is to minimize features that do not significantly contribute to the classification process. Table 5, Table 6 and Table 7 reveal that the accuracy values of the two-class classification and multi-class classification are relatively similar. Even with different window lengths or classification models, the accuracy of the original, SFS, and SBS do not differ too much. However, in Table 8, the number of features after using SFS and SBS is greatly reduced. In practical applications, if the number of input data per class is huge, then a small number of features can aid in substantial computation. In the SFS method, there are four features with essential contributions in all four windows (10-/20-/30-/60-sec), namely F1, F9, F10, F20, and F25. In the SBS method, the number of features selected in all four windows increases significantly. Most of the features extracted from ***D1*** and ***D2*** are generally preferred. The number of features contributing to this approach are F1, F9-10, F40-41, F49, F57, F59-60, and F64-72. With the detailed investigation, the most selected features are MSE features. It demonstrates an essential contribution of MSE features in the training process and the improved accuracy of the proposed algorithm

### 4.5. Comparison with Existing Studies

The main contribution of this study can be found by comparing the existing literature using the same database [9]. Table 9 reveals the classification results of the proposed algorithm comparing to other literature [11,12,13,14,15,16], the time window length of 10-sec with the KNN model from the proposed algorithm are used to compare with other literature. For the classification of the HC group and each disease from NDD groups, the proposed algorithm outperforms or equal to the performance to that of the [11,12,13,14,15,16]. For classification of any two disease groups from NDD groups, the performance of this study outperforms that of the [11,12,13,16] but little less than [14]. However, the accuracy is less than 0.5%. For the classification of the HC and each disease in the NDD groups, only this study and [16] had reported the accuracy. The proposed algorithm can achieve the accuracy of 99.56%/99.68% under without/with SMOTE data augmentation, which is better than the accuracy reported by [16] (97.87%).

## 5. Conclusions

In this paper, an NDD gait classification algorithm based on the differential transformation of GF signal and MSE values combined with statistical values was proposed. Moreover, the accuracy of the proposed algorithm also improved by applying the SMOTE method to balance the amount of data in each class. Sequential feature selection methods successfully to reduce the number of non-essential features while maintaining accuracy and reduced the training time of classification models. Finally, KNN and SVM models were used to classify HC and NDD and obtained satisfactory classification results. This study successfully developed an NDD gait classification algorithm using MSE and machine learning classifier.

## Figures and Tables

**Table 1:** Summary of neurodegenerative disease (NDD) gait classification articles.

Summary of the NDD Gait Classification in Existing Articles			
Articles	Features Extraction	Classification Model	Validation
[11]	Biometric features	SVM and the radial basic function kernel	10-fold cross-validation
[12]	Biometric features, features from time and frequency domain	Quadratic Bayes normal classifier, SVM	LOOCV
[13]	Statistical values	SVM, MLP, KNN	LOOCV
[14]	Parameters of RQA, statistical values	SVM, PNN	LOOCV
[15]	Fuzzy recurrence plot Gray-level Co-occurrence matrix	LS-SVM and LDA	LOOCV
[16]	Recurrence plot, PCA	CNN (Alexnet)	LOOCV

Notes: RQA: recurrence quantification analysis, FRP: fuzzy recurrence plot, GLCM: gray level co-occurrence matrix, PCA: principal component analysis, LS-SVM: Least squares support vector machines, LDA: linear discriminant analysis.

**Table 2:** Demographics of the subjects in the neurodegenerative disease database.

Class	Number	Ages (Year)	Weight (kg)	Gait Speed (m/s)
ALS	13	66.8 ± 10.85	77.11 ± 21.15	1.05 ± 0.22
PD	15	46.65 ± 12.6	75.07 ± 16.9	1.0 ± 0.2
HD	20	55.62 ± 12.83	73.47 ± 16.23	1.15 ± 0.35
HC	16	39.31 ± 18.51	66.81 ± 11.08	1.35 ± 0.16

**Table 3:** Feature description after calculating the mean, standard deviation, and multiscale sample entropy.

Feature Notation	Feature Description
F($8\times \left(i-1\right)+1$)	Mean values
F($8\times \left(i-1\right)+2$)	STD values
F($8\times \left(i-1\right)+2+s$)	MSE (*s* = 1–6) values
where *i* = {1, 2, 3,...,9} corresponds to LF/RF/AF/LF1/RF1/AF1/LF2/RF2/AF2	

**Table 4:** Number of initially extracted samples (IES), verified samples (VS), and after using SMOTE.

Class	Number of Gait Force Signals											
	10-sec *d* = 5, *TW* = 10			20-sec *d* = 10, *TW* = 20			30-sec *d* = 15, *TW* = 30			60-sec *d* = 30, *TW* = 60		
	IES	VS	SMOTE	IES	VS	SMOTE	IES	VS	SMOTE	IES	VS	SMOTE
ALS	715	690	1093	351	321	539	229	206	340	108	98	160
PD	825	803	1096	405	381	540	265	241	340	125	104	160
HC	880	856	1094	432	417	540	282	261	340	132	110	160
HD	1100	1097	1097	540	540	540	353	340	340	166	160	160

**Table 5:** Classification result summary for two-class classification of HC and NDD using 10-fold cross-validation for 10-, 20-, 30-, and 60-sec time window lengths.

Model	SMOTE	Feature Selection	HC vs. PD				HC vs. HD				HC vs. ALS			
			10-sec	20-sec	30-sec	60-sec	10-sec	20-sec	30-sec	60-sec	10-sec	20-sec	30-sec	60-sec
KNN	Without	All Features	100%	100%	98.24%	97.06%	99.9%	100%	99.67%	98.96%	99.94%	100%	98.76%	97.37%
	With	All Features	99.90%	100%	98.95%	98.20%	99.80%	100%	99.55%	99.40%	100%	100%	99.10%	99.10%
	With	SFS Features	99.90%	99.90%	99.10%	99.40%	99.80%	100%	98.65%	99.70%	100%	100%	98.95%	99.70%
	With	SBS Features	99.85%	100%	98.65%	99.40%	99.85%	100%	98.85%	99.70%	99.95%	100%	99.55%	99.70%
SVM	Without	All Features	99.82%	99.75%	99.41%	97.50%	99.65%	99.49%	99.67%	98.61%	99.68%	99.74%	99.38%	97.81%
	With	All Features	99.85%	99.90%	99.55%	98.20%	99.85%	99.90%	99.55%	98.75%	100%	99.80%	99.85%	98.75%
	With	SFS Features	99.75%	99.90%	98.85%	99.10%	99.40%	99.10%	98.65%	98.40%	99.85%	99.70%	99.10%	99.10%
	With	SBS Features	99.80%	99.80%	99.40%	98.75%	99.70%	99.55%	99.40%	98.40%	99.90%	99.65%	99.70%	99.10%

**Table 6:** Classification result summary for two-class classification of each disease in NDD group using 10-fold cross-validation for 10-, 20-, 30-, and 60-sec time window lengths.

Model	SMOTE	Feature Selection	PD vs. HD				PD vs. ALS				HD vs. ALS			
			10-sec	20-sec	30-sec	60-sec	10-sec	20-sec	30-sec	60-sec	10-sec	20-sec	30-sec	60-sec
KNN	Without	All Features	99.84%	100%	98.62%	98.90%	99.93%	99.86%	97.32%	96.70%	99.49%	99.43%	98.36%	97.31%
	With	All Features	99.75%	100%	98.40%	99.60%	99.90%	100%	98.95%	97.80%	99.55%	99.50%	99.40%	98.20%
	With	SFS Features	99.80%	99.90%	98.65%	99.70%	99.90%	100%	99.25%	99.70%	99.65%	99.45%	99.10%	99.40%
	With	SBS Features	99.70%	99.90%	99.40%	99.70%	100%	100%	99.25%	100%	99.65%	99.45%	99.40%	99.40%
SVM	Without	All Features	99.57%	99.67%	98.96%	98.55%	99.79%	99.15%	99.33%	98.58%	99.83%	99.43%	99.45%	99.62%
	With	All Features	99.60%	99.50%	99.10%	98.40%	99.95%	99.70%	99.55%	99.10%	99.55%	99.60%	99.10%	98.20%
	With	SFS Features	99.15%	98.80%	98.25%	98.40%	99.55%	99.70%	98.70%	99.70%	99.30%	99.35%	98.65%	99.10%
	With	SBS Features	99.30%	99.15%	98.95%	98.20%	99.60%	99.20%	99.40%	99.10%	99.20%	99.10%	99.70%	98.75%

**Table 7:** The classification accuracy of HC, PD, HD, and ALS (multi-class classification).

Model	SMOTE	Feature Selection	HC vs. PD vs. HD vs. ALS			
			10-sec	20-sec	30-sec	60-sec
KNN	Without	All Features	99.56%	99.70%	96.98%	94.40%
	With	All Features	99.68%	99.77%	98.53%	96.41%
	With	SFS Features	99.73%	99.72%	98.53%	99.38%
	With	SBS Features	99.73%	99.77%	98.90%	99.69%
SVM	Without	All Features	99.27%	99.17%	99.15%	97.00%
	With	All Features	99.50%	99.44%	98.97%	98.13%
	With	SFS Features	99.04%	98.56%	97.64%	97.81%
	With	SBS Features	98.86%	98.70%	98.90%	97.34%

**Table 8:** Total number of selected features after the implementation of the sequential forward selection (SFS) and sequential backward selection (SBS) methods.

Feature Selection	Window Length	Number of Selected Features	List of Selected Features
SFS	10-sec	20	F1, F6, F7, F9-10, F15, F18, F20, F25, F27, F29, F31, F33, F35, F41, F43, F44, F47, F51, F67
	20-sec	13	F1, F2, F7, F9-10, F12, F20, F24-27, F49, F72
	30-sec	12	F1, F3, F10, F12-13, F21, F25, F37, F44, F51-52, F65
	60-sec	14	F1-4, F9-10, F20, F23-24, F36, F43, F46, F55, F57
SBS	10-sec	34	F1, F5, F7, F9-10, F14-15, F21, F24-25, F28, F30, F32, F33-34, F36, F40-41, F49-50, F52, F57-60, F62-F68, F70-72
	20-sec	18	F1, F6, F8-10, F21, F41, F44-45, F49, F55, F60, F63, F66, F69-72
	30-sec	48	F1-5, F9, F12, F14, F16, F18, F20, F22, F26, F28, F36-37, F39-60, F62, F64-72
	60-sec	24	F10, F12, F18, F24, F29, F39, F40, F46-48, F53, F56-57, F59-F61, F64-65, F67-F72

**Table 9:** Accuracy comparison between the proposed work and existing literatures using NDD database [9].

Classification Task	[11]	[12]	[13]	[14]	[15]	[16]	Proposed Algorithm without SMOTE	Proposed Algorithm with SMOTE
HC vs. PD	86.43%	85.89%	100%	100%	100%	100%	100%	99.90%
HC vs. HD	84.17%	85.32%	100%	100%	100%	98.41%	99.90%	99.80%
HC vs. ALS	93.96%	93.86%	96.55%	96.15%	100%	100%	99.94%	100%
PD vs. HD	79.04%	79.48%	91.18%	100%	-	97.25%	99.84%	99.75%
PD vs. ALS	85.47%	85.09%	96.43%	100%	-	95.95%	99.93%	99.90%
HD vs. ALS	86.52%	84.78%	96.88%	100%	-	100%	99.49%	99.55%
HC vs. PD vs. HD vs. ALS	-	-	-	-	-	97.87%	99.56%	99.68%
